# Unveiling the Link Between COVID‐19 and Pulmonary Hypertension

**DOI:** 10.1155/cjid/9352258

**Published:** 2026-03-31

**Authors:** Jad Abdul Khalek, Mohamad Al Hajjar, Zeina Al-Khalil, Jana Salem, Rana Zareef, Fadi Bitar, Mariam Arabi

**Affiliations:** ^1^ Faculty of Medicine, American University of Beirut Medical Center, Beirut, Lebanon, aubmc.org.lb; ^2^ Department of Pediatrics and Adolescent Medicine, American University of Beirut, Beirut, Lebanon, aub.edu.lb; ^3^ Division of Pediatric Cardiology, Department of Pediatrics and Adolescent Medicine, American University of Beirut Medical Center, Beirut, Lebanon, aubmc.org.lb

**Keywords:** acute respiratory distress syndrome, COVID-19, inflammatory markers, pulmonary hypertension, vascular inflammation

## Abstract

**Introduction:**

The COVID‐19 pandemic, caused by the SARS‐CoV‐2 virus, was first identified in Wuhan, China, in December 2019 and has had a significant impact on global health. The virus uncovered multifaceted complications across various organ systems, including the cardiovascular system. Pulmonary hypertension, often exacerbated in settings of severe or prolonged viral infections, presents unique challenges in the context of COVID‐19 due to its complex pathophysiology involving vascular inflammation and thrombotic events.

**Methods:**

This narrative review aims to delineate the emerging relationship between COVID‐19 and pulmonary hypertension, focusing on pathophysiological insights, clinical implications, and evolving therapeutic strategies, enriching clinical practice and guiding future research of this complex interaction. We conducted a comprehensive literature review using databases such as MEDLINE, PubMed, and Google Scholar with keywords related to pulmonary hypertension and COVID‐19, covering studies published between December 2019 and February 2025.

**Results:**

COVID‐19 has been linked to an increased incidence of pulmonary hypertension due to direct viral effects on pulmonary vasculature and secondary inflammatory responses. Clinical manifestations of pulmonary hypertension in COVID‐19 include typical symptoms such as dyspnea and chest pain. Effective management strategies include the use of vasodilators, anticoagulants, and tailored experimental treatments. The review also emphasizes the importance of long‐term follow‐up and monitoring to evaluate disease progression and treatment response.

**Conclusion:**

The intersection of COVID‐19 and pulmonary hypertension presents a significant challenge in cardiovascular medicine, necessitating advanced diagnostic and therapeutic approaches. The insights gained from the current pandemic will likely influence broader public health strategies and clinical protocols, improving outcomes for patients with pulmonary hypertension and those at risk of severe viral infections. Future research should focus on personalized medicine approaches and the development of innovative treatments to manage the unique aspects of pulmonary hypertension in the context of COVID‐19.

## 1. Introduction

The dreaded COVID‐19 pandemic caused by the novel severe acute respiratory syndrome coronavirus 2 (SARS‐CoV‐2) took the world by storm. This virus was first discovered in Wuhan, China, in December 2019, and it profoundly impacted global health [1]. The virus was originally recognized for its severe respiratory side effects. The coronavirus unraveled its capacity to adversely affect many organ systems, including the cardiovascular system [2]. Among the plethora of complications caused by COVID‐19, pulmonary hypertension (PH) is an often unrecognized consequence, particularly in patients with severe or long‐term infections [3]. PH is defined as an elevation of mean pulmonary artery pressure to above 20 mm Hg (*mPAP* ≥ 20 mmHg), which imposes an increased workload on the right ventricle, leading to eventual right heart failure if not managed effectively [4].

Historically, PH has been linked to conditions like chronic obstructive pulmonary disease (COPD), congenital heart disease (CHD), and pulmonary embolism (PE) [5–7]. However, the potential of viral illnesses to induce PH has been revealed by COVID‐19 [8]. The underlying mechanisms for this association are intertwined and involve inflammatory responses, viral effects on the pulmonary vasculature, and thrombotic events that can increase pulmonary vascular resistance [9]. The virus’s ability to trigger a series of events, including dysfunction of the endothelium and long‐term inflammation, exacerbates pulmonary vascular resistance [10].

PH patients with COVID‐19 often exhibit subtle and nonspecific symptoms such as dyspnea, fatigue, and chest pain, which overlap with the usual COVID‐19 symptoms [11]. COVID‐19 can also lead to other complications like acute respiratory distress syndrome (ARDS) and heart failure, which may clinically resemble PH [12, 13]. Prompt recognition and accurate differentiation are crucial for timely diagnosis and proper treatment. Strategies for treating PH in COVID‐19 patients include using vasodilators, anticoagulation therapy, and experimental treatments explicitly tailored for COVID‐19 patients [14, 15].

Long‐term follow‐up is essential for COVID‐19 patients with PH, as it helps monitor disease progression and treatment efficacy. Follow‐up protocols typically include regular imaging studies, functional assessments, and monitoring of biomarkers [16, 17]. This comprehensive care approach is vital to managing potential complications that can decrease long‐term adverse effects. This narrative review aims to provide an overview of the emerging relationship between COVID‐19 and PH, offering insights that will enhance clinical practice and direct future research in dealing with complex pandemics.

## 2. Methods

**FIGURE 1:**
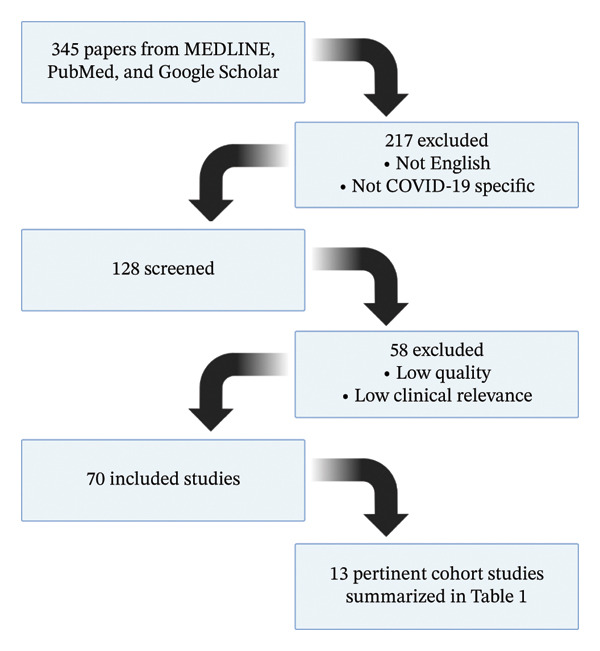
Study selection flow diagram.

A comprehensive literature search was conducted on February 5, 2025, utilizing three databases, MEDLINE, PubMed, and Google Scholar, to explore the relationship between PH and COVID‐19. The search employed specific keywords, including “pulmonary hypertension,” “COVID‐19,” “echocardiography,” “right heart catheterization,” “treatment outcomes,” “inflammatory markers,” and “vascular remodeling.” The inclusion criteria included English language, peer‐reviewed publications (randomized controlled trials, observational studies, meta‐analyses, case reports, and review articles) that focused on the relationship between PH and COVID‐19 and were published between December 2019 and February 2025. Exclusion criteria included studies that were not published in English, did not specifically address PH or COVID‐19, and lacked relevant clinical data on the pathophysiology or treatment of PH in the context of COVID‐19. The screening process involved reviewing the titles, abstracts, and full texts to ensure alignment with the review’s objective. The selection process is visually represented by Figure 1.

## 3. Results

### 3.1. Pathophysiology of PH in COVID‐19

Lung autopsies of patients who died from COVID‐19 have shown severe endothelial damage and thrombosis in pulmonary vessels. Additionally, consistently elevated levels of D‐dimer, a marker of thrombosis, were observed in COVID‐19 patients [18]. This procoagulant picture is particularly prevalent in the segmental pulmonary arteries. Therefore, it is hypothesized that the pulmonary arterial thrombosis seen in COVID‐19 patients is mainly due to the in situ inflammation‐driven vasoconstriction and thrombosis accompanying the infection, rather than a migrating deep vein thrombus (DVT). A systematic review and meta‐analysis by Suh et al. revealed that more than half of COVID‐19 patients with a PE lacked a DVT, and most PEs were located in the distal pulmonary vasculature [19]. Although it is well recognized that infection and inflammation drive a hypercoagulable state in the body, the COVID‐19 virus appears to promote localized thrombin generation and deposition, with microangiopathy, further increasing the risk of pulmonary vascular complications [20].

COVID‐19 uses angiotensin‐converting enzyme‐2 (ACE‐2) as a receptor. ACE‐2 is expressed in the lungs, blood vessels, and other tissues. It is hypothesized that the binding of the virus to endothelial ACE‐2 may trigger an inflammatory response and increase the risk of micro‐ and macrovascular thrombosis; this might explain the heightened incidence of thrombotic events in COVID‐19 patients [18, 21, 22]. Additionally, there is evidence that severe COVID‐19 is due to an exaggerated immune response often accompanied by an IL‐6‐triggered cytokine storm; thus, both lung injury and hypercoagulability occur secondary to the hyperinflammatory state. Endothelial damage and activation of the coagulation cascade will lead to pulmonary artery thrombosis, which—especially in patients with COVID‐19 pneumonia—can be exacerbated by pulmonary hypoxia, culminating in a vicious cycle [20]. Hypoxia itself is a significant element underlying thrombosis in COVID‐19. Hypoxia‐inducible transcription factors (HIFs) are released in response to hypoxia and play a role in the vascular response to hypoxia. HIFs are known to promote thrombosis and cellular inflammation by way of plasminogen activator inhibitors, tissue factors, and inhibition of thrombomodulin [18].

**FIGURE 2:**
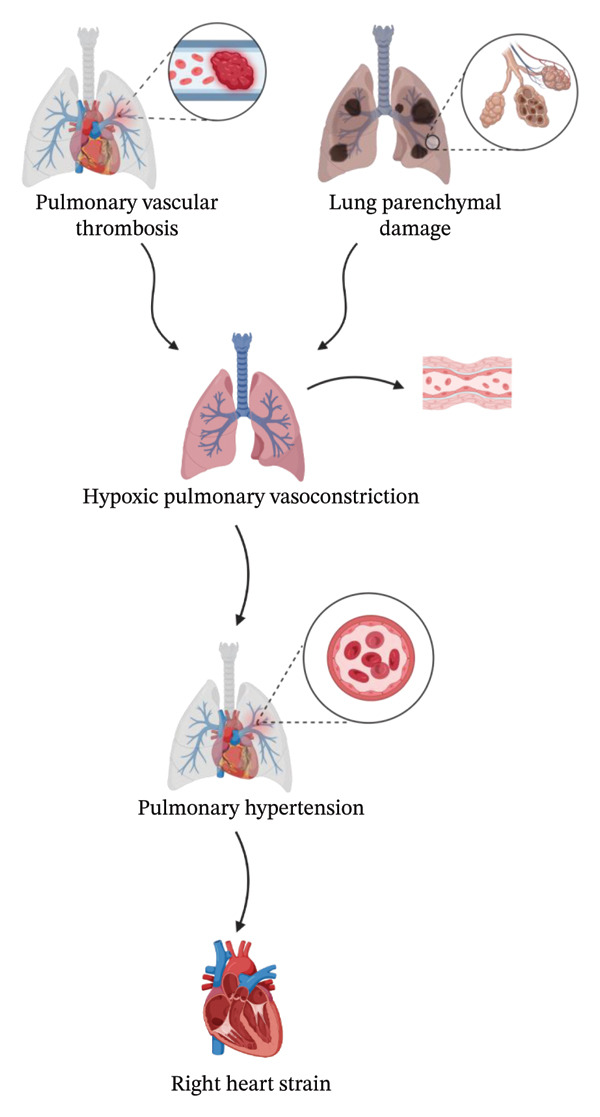
Pulmonary hypertension pathophysiology: This figure illustrates the progressive pathway from initial lung injury to the development of pulmonary hypertension. Lung parenchymal damage can occur due to various factors such as pneumonitis or pneumonia, which can lead to pulmonary vascular thrombosis, where blood clots form within the pulmonary vasculature, obstructing blood flow and increasing vascular resistance. Subsequent hypoxic pulmonary vasoconstriction, a reflexive narrowing of blood vessels in response to low oxygen levels, further elevates pulmonary arterial pressure. This chronic state of hypoxia and increased pressure places undue strain on the right ventricle of the heart, depicted here as right heart strain.

Lung parenchymal damage and pulmonary vascular thrombosis may lead to PH and right heart strain, mainly because of hypoxic pulmonary vasoconstriction. These mechanisms of action have been highlighted in Figure 2. The use of positive end‐expiratory pressure in mechanical ventilation of COVID‐19 patients also appears to influence pulmonary vascular hemodynamics. PH is associated with increased ICU admission and mortality [21, 23]. Moreover, it is well‐known that PH is a root cause of right ventricular (RV) dysfunction, which manifests on echocardiography as RV dilatation and hypokinesis [24]. Argulian et al. studied the link between RV size and in‐hospital mortality in COVID‐19 patients. Among 105 patients, 41% of deaths were observed in those with RV dilatation, with RV enlargement being the only statistically significant variable associated with in‐hospital mortality (odds ratio: 4.5; 95% confidence interval: 1.5–13.7; *p* = 0.005) [25]. RV enlargement and/or hypokinesis is likely to have been present in some of these patients prior to infection, making RV dysfunction a poor prognostic factor in hospitalized COVID‐19 patients and not necessarily a sequela of infection. Furthermore, the mechanism of RV dilatation in this cohort is likely multifactorial. Computed tomography angiography (CTA) of the chest was obtained in 31% of patients, half of whom showed evidence of PE. In COVID‐19 survivors, persistent evidence of RV systolic dysfunction in association with PH has been described, highlighting the importance of dedicated cardiopulmonary follow‐up. PH and its effects on cardiac function have been studied extensively in the literature, with various studies showcasing increased rates of PH and right heart strain in COVID‐19 patients. A more recent study by Rossi et al. investigated the “post‐COVID syndrome” in 25 patients hospitalized for COVID‐19 between March 2020 and February 2021. All patients in this study who presented with symptoms of fatigue and tachycardia postdischarge were found to have some degree of PH and severe RV systolic dysfunction, with statistically significant differences in echocardiography parameters between symptomatic and asymptomatic patients [26]. Whether these changes are reversible remains a topic for future discussion.

### 3.2. Clinical Presentation

PH has become a notable complication for patients recovering from COVID‐19, manifesting with persistent and progressive cardiovascular symptoms that can complicate the recovery process. The clinical presentation of PH in these patients is characterized by persistent dyspnea, fatigue, and chest pain, which do not improve with the resolution of other COVID‐19 symptoms [27].

Dyspnea is particularly troubling because it occurs even at rest or with minimal exertion and does not diminish as the acute respiratory symptoms of COVID‐19 resolve. The persistence of dyspnea can affect daily activities and quality of life, making it a key indicator of potential underlying issues such as PH. Patients often report fatigue that is disproportionate to their activity levels [28]. This fatigue is not only a direct consequence of PH but also reflects the overall burden of the disease on the cardiovascular system, contributing to the complexity of patient management. The chest pain associated with PH can be misleading, as it might mimic other cardiac conditions. It typically presents as a sharp or pressing pain, exacerbated by physical activity or deep breathing, and can further complicate the clinical differentiation between ongoing post‐COVID complications and other potential cardiac issues [29]. These symptoms, combined with signs of right heart strain such as edema and palpitations, paint a clinical picture that necessitates thorough evaluation [30]. The persistence of these symptoms, particularly when they do not align with the general trajectory of recovery from COVID‐19, should prompt consideration of PH among the differential diagnoses.

### 3.3. Diagnosis

COVID‐induced PH is initially suspected based on clinical examination and ECG findings, but the diagnosis is only confirmed through echocardiography and cardiac catheterization [31]. PH is defined as a mean pulmonary artery pressure ≥ 20 mmHg at rest, measured through right heart catheterization [32]. This method remains the most accurate for diagnosing PH [33]. However, due to the emergent nature of COVID‐19 cases, transthoracic echocardiography (TTE) has become a more efficient diagnostic tool [34]. Echocardiography, using Doppler techniques, can measure tricuspid regurgitation velocity peak, right atrial pressure, and systolic pulmonary artery pressure and can reveal signs indicative of PH [35].

In COVID‐19 patients, computed tomography (CT) scans are key in revealing characteristic pulmonary changes, including ground‐glass opacifications and consolidations [36]. These imaging findings, while typical in many viral pneumonias, are particularly prevalent in severe COVID‐19 cases. CT scans are not routinely used in all COVID‐19 patients but are reserved for those with severe symptoms or complications where detailed pulmonary imaging is necessary [37]. If echocardiography indicates potential PH, a CT scan may be done to assess the extent of pulmonary involvement. This is especially pertinent in high‐risk patients or those suspected of having concurrent complications such as PE, where CTA can provide critical insights into pulmonary arterial and venous systems [38].

Right heart catheterization is another diagnostic tool employed in severe cases of COVID‐19‐associated PH. It is used for precise monitoring of volume status, vascular resistance, and cardiac output, as well as for the direct measurement of pulmonary artery pressures [39]. This procedure is particularly valuable in patients receiving mechanical ventilation or extracorporeal membrane oxygenation (ECMO), as these interventions can significantly alter cardiovascular dynamics [40]. By providing detailed hemodynamic data, right heart catheterization helps tailor treatment strategies, including adjusting ventilatory settings and optimizing circulatory support, ensuring targeted therapeutic interventions.

### 3.4. Treatment

Targeted management of PH following COVID‐19 is essential for enhancing patient outcomes [41]. Pharmacological therapies for PH include the prostacyclin class, prostacyclin analogs, endothelin receptor antagonists (ERAs), nitric oxide, phosphodiesterase‐5 inhibitors (PDE‐5 inhibitors), and combinations of these preparations [42].

Inhaled nitric oxide has demonstrated positive outcomes by reducing systolic pulmonary artery pressure, improving systemic oxygenation, and preventing RV failure [43]. It acts as a vasodilator, diffusing into adjacent vascular smooth muscle to activate the cGMP pathway, which reduces muscular tone and promotes muscle relaxation. Additionally, nitric oxide has anti‐inflammatory properties, inhibits smooth muscle proliferation, and prevents platelet aggregation [44]. It enhances oxygenation by improving ventilation–perfusion (V/Q) matching and selectively dilating well‐ventilated lung areas. Furthermore, studies have shown that inhaled nitric oxide inhibits SARS‐CoV‐2 viral replication, potentially preventing disease progression. Some of the proposed mechanisms include interference with target receptor binding and fusion with host cells, as well as suppression of cysteine proteases involved in viral RNA replication [45].

PDE‐5 inhibitors, such as sildenafil and tadalafil, have long been used to treat PH. These potent, nonselective vasodilators work by extending the action of cGMP through the nitric oxide pathway. PDE‐5 inhibitors can mitigate the effects of angiotensin type I receptors, lowering proinflammatory cytokines and reducing inflammatory cell infiltration in the alveoli [46]. Additionally, they prevent the transformation of endothelial and smooth muscle cells in the pulmonary artery into mesenchymal cells, reducing the risk of clotting and thrombotic complications while enhancing gas exchange [47]. A recent study published in May 2023 showed that sildenafil treatment may lower the risk of death, the likelihood of intubation, and the length of ICU stay for patients with severe COVID‐19 and pulmonary arterial hypertension [48].

ERAs such as Bosentan, which counteract endothelin‐1, a powerful vasoconstrictor, may also help prevent lung injury associated with COVID‐19 due to their anti‐inflammatory properties [49]. High levels of endothelin‐1, which are highly expressed in PH and are active proinflammatory vasoconstrictors, may exacerbate lung injury in COVID‐19 [50]. Therefore, ERAs might be beneficial for patients with PH. However, some studies show higher long‐term mortality and oxygen therapy requirements after Bosentan use [51].

Prostacyclin, like Epoprostenol, is another treatment option for PH. Similar to nitric oxide, it induces smooth muscle relaxation and prevents platelet aggregation [52]. Additionally, prostacyclin has antiproliferative and cytoprotective functions. Inhaled prostacyclin analogs like iloprost and treprostinil are a cornerstone in the therapy of PH [53, 54]. Two clinical studies have reported statistically significant cardiovascular and pulmonary improvements in severely infected COVID‐19 patients treated with these analogs [55, 56].

Novel treatments targeting endothelial dysfunction, inflammation, and thrombosis may enhance COVID‐19‐associated PH outcomes. Gene and cell‐based therapeutics for reversing vascular remodeling are being investigated [57]. Mesenchymal stem cell (MSC) treatment, which has shown promise in rebuilding damaged pulmonary vasculature, is a potential breakthrough [58]. Furthermore, investigational medications that target the bone morphogenetic protein receptor type II (BMPR2) pathway could help prevent pulmonary arterial remodeling [59].

### 3.5. Follow‐Up

A structured follow‐up approach for post‐COVID‐19 patients with PH is proposed to address the potential for both lung fibrosis and thromboembolic pulmonary vascular complications [60, 61]. Central to this algorithm is perfusion imaging, which plays a critical role in assessing pulmonary vascular sequelae. The inclusion of perfusion imaging is recommended when abnormalities are identified through lung function tests, exercise physiology assessments, or TTE, regardless of whether thromboembolism was documented during the acute phase of the illness [60, 62]. Two key lung perfusion imaging modalities are emerging as crucial tools: dual‐energy computed tomography (DECT) and VQ scintigraphy, now advanced to 3D single‐photon emission computed tomography with CT fusion (SPECT–CT) [60]. This modern VQ SPECT–CT approach, enhanced with CT lung overlays, provides greater diagnostic accuracy compared to traditional techniques. DECT perfusion imaging has demonstrated similar patterns, capturing both venous thromboembolism and small vessel angiopathy during acute severe COVID‐19 pneumonia [60]. CT scans are recommended primarily for patients with severe COVID‐19 disease or persistent symptoms postrecovery, aligned with pneumonia management guidelines, where imaging is offered to those symptomatic or at cancer risk [63–65]. While perfusion imaging methods such as DECT and V/Q SPECT–CT provide a proper understanding of post‐COVID pulmonary vascular sequelae, the existing information is mostly derived from observational studies and single‐center cohorts, limiting generalizability. In addition, perfusion imaging is increasingly included in follow‐up algorithms; however, the optimal timing, patient selection, and prognostic importance of these data are still not fully understood.

According to a study published in 2024, functional assessments of patients with PH after COVID‐19 were divided into three subgroups based on available follow‐up data [66]. The first subgroup involved evaluating WHO Functional Class (WHO‐FC), 6‐min walk test (6‐MWT) distances, and brain natriuretic peptide (BNP) levels. The 6‐MWT was conducted according to American Thoracic Society (ATS) standards, offering a standardized measure of exercise tolerance. The second subgroup focused on pulmonary function tests (PFT) with diffusing capacity for carbon monoxide (DLCO) [66, 67]. DLCO was measured using the single‐breath method, with the lower limit of normal (LLN) defined as the fifth percentile based on Global Pulmonary Function Initiative reference values [66]. In addition, a retrospective study of 23 pediatric PH patients diagnosed with COVID‐19 at Texas Children’s Hospital revealed significant functional impacts. Post‐COVID functional assessments showed notable declines in physical and pulmonary function [68]. PFT data indicated decreased predicted forced vital capacity (FVC) by a median of 6% (range −11% to +6%) and forced expiratory volume in one second (FEV1) by a median of 14% (range −12% to −18%) in 75% of tested patients [68].

### 3.6. A Focus on the Pediatric Population

**FIGURE 3:**
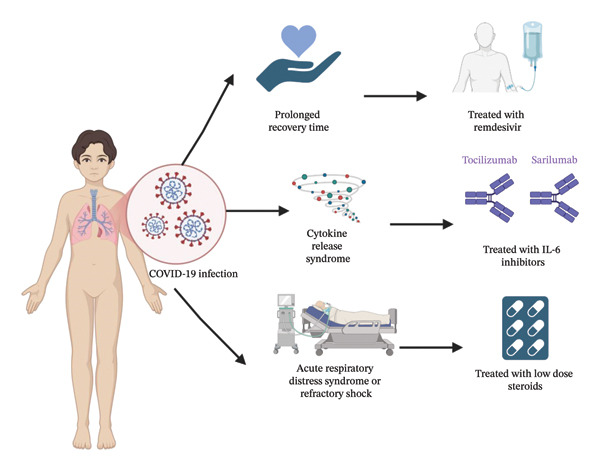
Treatment regimens of severe COVID‐19 symptoms presenting in the pediatric population.

The management of PH in children in the era of the COVID‐19 pandemic requires a structured and multidisciplinary approach. Any child with existing or newly diagnosed PH presenting with fever, respiratory distress, or hypoxemia should be tested for SARS‐CoV‐2 [69]. If the antigen test is negative but clinical suspicion for COVID‐19 remains, antibody screening is recommended. A thorough evaluation, including a detailed history of COVID‐19 exposure, physical examination, and laboratory tests such as chest X‐ray, electrocardiogram, C‐reactive protein, D‐dimer, lactate dehydrogenase, procalcitonin, brain natriuretic peptide, and troponin, is essential [69, 70]. While no specific treatments have been proven effective for severe COVID‐19 in children with PH, European guidelines suggest using low‐dose steroids for severe cases, such as those with refractory shock or ARDS requiring mechanical ventilation [69, 70]. Remdesivir may shorten recovery time in severe cases, and therapies targeting hyperinflammation, such as IL‐6 inhibitors (tocilizumab or sarilumab) and IL‐1 receptor antagonists (anakinra), may be beneficial for managing cytokine storm syndrome [69]. Treatment for pediatric COVID‐19 complications is graphically represented in Figure 3. While these strategies provide a practical foundation for therapy, evidence for the efficacy of immunomodulatory medications in this population is scarce, emphasizing the importance of specialized pediatric trials.

### 3.7. Cohort Studies Related to PH and COVID‐19

Table 1 highlights and summarizes pertinent cohort studies examining various complications of COVID‐19 and PH. These cohort studies were published between 2022 and 2024 and were retrieved from PubMed and MEDLINE.

**TABLE 1:** Comprehensive cohort studies related to PH and COVID‐19 over the years 2022–2024.

Study	Study year	Study type	Patient number	Age (years) (mean or median)	Key outcomes
Impact of the COVID‐19 Pandemic on Pulmonary Hypertension Patients: Insights from the BNP‐PL National Database [8]	July 2022	National Database Review	1704 patients	55.7 y (mean)	The pandemic caused decrease in diagnosis rates and escalation of therapy and an increase in pulmonary hypertension mortality.
Pulmonary Artery Pressures and Right Ventricular Dimensions of Post‐COVID‐19 Patients Without Previous Significant Cardiovascular Pathology [71]	August 2022	Retrospective cohort	91 patients	49 y (median)	Patients without a history of pulmonary hypertension and no risk factors presented for cardiac complaints in the outpatient department showed to have elevated pulmonary artery pressure and right heart dimensions. 48 patients had severe pulmonary involvement, and 21 presented with nonsevere pulmonary involvement.
COVID‐19 in Patients with Pulmonary Hypertension: A National Prospective Cohort Study [70]	September 2022	Prospective Cohort	211 patients	63.9 y (median)	High mortality among patients with precapillary pulmonary hypertension.
Severe Covid‐19 and Acute Pulmonary Hypertension: 24‐Month Follow‐Up Regarding Mortality and Relationship to Initial Echocardiographic Findings and Biomarkers [72]	November 2022	Retrospective Cohort	67 patients	57 y (median)	Increased mortality risk up to 24 months post‐transthoracic echocardiography diagnosis of arterial pulmonary hypertension.
Pulmonary Hypertension at Admission Predicts ICU Mortality in Elderly Critically Ill With Severe COVID‐19 Pneumonia: Retrospective Cohort Study [73]	January 2023	Retrospective cohort	117 patients	77 y (mean)	Elevated pulmonary hypertension at the time of admission is an independent predictor of intensive care unit and hospital mortality in elderly patients with severe COVID‐19 pneumonia.
Abnormal Right Ventricular Echocardiographic Findings in Recovered Patients Associated With Severe Acute Respiratory Syndrome in COVID‐19 [74]	February 2023	Retrospective cohort	61 patients	54.2 y (mean)	Severe acute respiratory syndrome is associated with abnormal right ventricular echocardiographic findings in patients who have recovered from COVID‐19.
Trends in COVID‐19‐Associated Mortality in Patients With Pulmonary Hypertension [75]	March 2023	Retrospective Analysis	253 patients	64 y (median)	Reported a decline in COVID‐19‐associated mortality among pulmonary hypertension patients with the emergence of Omicron variants.
The Outcome of Patients with Pulmonary Arterial Hypertension and Chronic Thromboembolic Pulmonary Hypertension During the COVID‐19 Pandemic [76]	June 2023	Cross‐sectional study	75 patients	49 y (mean)	Pulmonary hypertension Patients with COVID‐19 infection seems to be associated with high mortality and morbidity.
Pulmonary Hypertension Outcomes During the COVID‐19 Pandemic in Brazil [77]	November 2023	Retrospective Analysis	272 patients	54 y (mean)	The report underscores that while the incidence of COVID‐19 in pulmonary hypertension patients mirrors that of the general population in Brazil, the case‐fatality rate is significantly higher, with implications for patient care in low‐ and middle‐income countries as new COVID‐19 variants emerge.
Chronic Thromboembolic Pulmonary Hypertension after Pulmonary Embolism in SARS‐CoV‐2 [78]	February 2024	Prospective observational	133 patients	65.5 y (mean)	Thrombotic defects persist after pulmonary embolism in 18% of patients post‐SARS‐CoV‐2 infection, yet the incidence of chronic thromboembolic pulmonary hypertension is lower at 0.75% in COVID‐19–related pulmonary embolism compared to pulmonary embolism not related to COVID‐19.
Patients With Pulmonary Arterial Hypertension and Chronic Thromboembolic Pulmonary Hypertension During the COVID‐19 Pandemic [79]	March 2024	Clinical Outcomes Study	88 patients	58 y (median)	This study reveals that patients with pulmonary hypertension faced a reduced risk of severe COVID‐19 following the emergence of the Omicron variant, despite experiencing multiple infections and reinfections, even though they were vaccinated.
Vaccination Against Coronavirus Disease 2019 in Patients With Pulmonary Hypertension [80]	March 2024	Prospective Cohort	706 patients	40.3 y (mean)	Found COVID‐19 vaccination safe for pulmonary hypertension patients, no increase in pulmonary hypertension‐related adverse events.
The Impact of COVID‐19 Infection on Patients with Severe Chronic Pulmonary Hypertension [66]	April 2024	Prospective Cohort	51 patients	58 y (mean)	Low overall mortality, some cases worsening heart failure post‐infection

## 4. Discussion

PH induced by COVID‐19 is prevalent. Recent observational studies have reported an incidence of approximately 12%–13% for signs of PH detected via echocardiography in hospitalized COVID‐19 patients [81]. In a study of 245 patients listed for lung transplantation due to COVID‐19–related lung disease, 27.9% were identified with precapillary PH [82].

**FIGURE 4:**
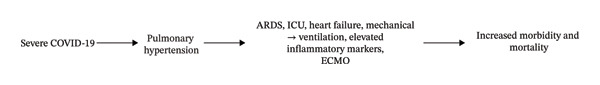
Severe COVID‐19 infection flowchart representing the severe effects of COVID‐19.

Various mechanisms account for RV dysfunction, vascular thrombosis, and PH in the setting of COVID‐19 infection. Figure 4 represents the effects of severe COVID‐19 infection. Severe COVID‐19 may contribute to the development of PH or exacerbate pre‐existing pathology, which results in increased morbidity and mortality through a domino‐like effect. Patients with severe COVID‐19 and PH, therefore, experience poorer clinical outcomes [83].

Emerging therapies for PH in the context of COVID‐19 focus on addressing the complex pathophysiological mechanisms triggered by the virus. Innovative treatments target the unique aspects of PH that resemble cancerous processes, such as excessive cell proliferation and resistance to apoptosis. One promising approach involves the repurposing of cancer drugs that inhibit proliferative pathways and regulate mitochondrial dysfunction. Drugs like elamipretide, which target mitochondrial metabolism and reduce oxidative stress, are currently under investigation [42]. Similarly, agents like dichloroacetate are being explored to shift the metabolic balance from glycolysis back to oxidative phosphorylation, potentially reversing the Warburg effect observed in pulmonary vascular cells during PH [84].

Additionally, treatments that modulate the inflammatory response are gaining attention. Drugs that influence the inflammasome pathway, such as the IL‐1 receptor antagonist anakinra, are being repurposed to curb the excessive inflammatory response associated with PH [85]. Research continues to explore the use of other immunomodulatory drugs, reflecting a broadened understanding of PH as not only a hemodynamic disorder but also a disease of inflammation and metabolic dysregulation. The integration of these targeted therapies could lead to more effective management strategies tailored to the complexities of PH post‐COVID‐19, aiming to mitigate disease progression and improve patient outcomes.

Biomarkers play a crucial role in understanding the pathophysiology and prognosis of COVID‐19, particularly in patients with pulmonary complications such as hypertension. A study assessed cardiac biomarkers in COVID‐19 pneumonia patients with varying degrees of PH. Patients were categorized into three groups based on pulmonary artery systolic pressure: < 25 mmHg (Group 1), 25–40 mmHg (Group 2), and 40–60 mmHg (Group 3) [86]. Interestingly, NT‐proBNP levels were highest in Group 1, exceeding those in Groups 2 and 3 by 41.3% (*p* = 0.015) and 38.2% (*p* = 0.015), respectively. Conversely, Group 1 exhibited significantly lower nitrite (NO_2_) levels by 31.1% (*p* = 0.026) compared to Group 2 and by 62.8% (*p* = 0.008) compared to Group 3. Similarly, nitrate (NO_3_) levels were reduced by 28% (*p* = 0.029) and 54.6% (*p* = 0.006) compared to Groups 2 and 3, respectively. These findings suggest that severe COVID‐19 in patients with advanced PH is linked to disrupted nitrite and nitrate metabolism and lower NT‐proBNP levels [86]. Another study explored the gene expression profiles in human lung epithelial cells affected by SARS‐CoV‐2 infections, with a focus on pulmonary arterial hypertension as a complication in COVID‐19 patients [87]. Differential gene expression analysis identified several key biomarkers shared between SARS‐CoV‐2 infections and PH, including SAA2, S100A9, S100A8, SAA1, S100A12, and EDN1. Among these, S100A9 and S100A8 were highlighted for their significant roles in protein–protein interaction networks [87]. Collectively, these studies demonstrate that COVID‐19 may worsen PH through both hemodynamic stress and endothelial dysfunction, as seen by increased NT‐proBNP and disturbed nitrite/nitrate metabolism [86]. The discovered gene expression alterations, including elevation of inflammatory mediators such as S100A8 and S100A9, further suggest hyperinflammation and immune‐mediated vascular remodeling as possible mechanisms linking SARS‐CoV‐2 infection to PH development [87]. However, both studies are limited by small sample sizes, raising concerns regarding the link between these biomarkers and long‐term clinical outcomes.

The long‐term impact of COVID‐19 on pediatric patients with PH remains uncertain due to limited data [68]. Adult studies have shown post‐COVID PFT abnormalities, such as reduced total lung capacity, likely from deconditioning, and exertional hypoxemia [26]. In the context of long COVID, prevention of obesity in women is a particularly relevant issue that may influence pulmonary hemodynamics and long‐term cardiovascular outcomes [88]. While most pediatric PH patients have experienced favorable short‐term outcomes after COVID‐19, the potential long‐term consequences of the infection as a modifier of PH progression require ongoing monitoring and investigation [68, 69].

Although our narrative review provides a thorough and detailed examination of the existing literature on the impact of PH, it is important to acknowledge certain limitations. Despite extensive searches, our manuscript did not encompass all studies on the topic, as our literature search was confined to only three databases, potentially overlooking relevant studies. Moreover, the studies included primarily originate from countries within Europe and North America. This geographical limitation may introduce variability in disease burden, management practices, and patient outcomes, as these can differ significantly by region. Consequently, it was challenging to synthesize a globally representative overview from the available literature. Additionally, due to the scarcity of published research specific to certain aspects of PH, this review could not be restricted to studies from any single region, further complicating a uniform analysis. Moreover, longitudinal studies are needed to determine the extent and duration of PH and RV dysfunction after COVID‐19. The literature would benefit from prospective data exploring the rate of recovery of normal RV function and its relationship to post‐COVID symptoms.

## 5. Conclusion

In conclusion, exploring PH in the context of COVID‐19 presents a unique intersection of a global health crisis and a complex vascular condition. The studies reviewed highlight the multifaceted nature of PH in COVID‐19 patients, from its pathophysiology and clinical presentation to innovative treatment and the necessary follow‐up. While significant strides have been made in understanding and managing PH in COVID‐19, challenges remain. It is crucial to expand the scope of research to include diverse patient populations across different geographic regions. Much of the existing data comes from Europe and North America, and there is a significant gap in understanding how COVID‐19–related PH affects patients in other parts of the world. Future research should prioritize investigating the long‐term effects of COVID‐19 on pulmonary vascular health, including the durability of PH and right heart dysfunction post‐infection. Additionally, there is a need for personalized medicine approaches to better tailor therapies to individual patient characteristics. Research into novel therapies, such as gene therapy, stem cell treatments, and innovative drug classes targeting endothelial dysfunction, holds promise for improving outcomes in patients with COVID‐19–related PH. The ongoing evolution of pandemics and future crises makes adapting and refining therapeutic strategies crucial as new data becomes available. The lessons learned will undoubtedly influence future public health strategies and clinical protocols, ultimately leading to improved patient care and health outcomes worldwide.

## Author Contributions

Jad Abdul Khalek, Mohamad Al Hajjar, Zeina Al‐Khalil, Jana Salem, and Rana Zareef performed the search and wrote the first draft of the manuscript. Mariam Arabi conceived the presented idea and the framework. Rana Zareef, Fadi Bitar, and Mariam Arabi supervised the project and did the final editing. All authors contributed to corrections and adjustments of subsequent iterations of the manuscript.

## Funding

The authors received no specific funding for this work.

## Disclosure

Figures 1, 2, 3, and 4 were created on https://biorender.com. All authors approved and agreed with the content.

## Conflicts of Interest

The authors declare no conflicts of interest.

## Data Availability

Data sharing is not applicable to this article as no datasets were generated or analyzed during the current study.
